# Disease burden of breast cancer and risk factors in Europe 44 countries, 1990-2019: findings of the global burden of disease study 2019

**DOI:** 10.3389/fendo.2024.1405204

**Published:** 2024-05-23

**Authors:** Shaohong Yu, Xiuli Cai, Xinyu Wang, Xiuquan Lin, Shuanglong Cai

**Affiliations:** ^1^ Shengli Clinical Medical College of Fujian Medical University, Fuzhou, Fujian, China; ^2^ The School of Public Health, Fujian Medical University, Fuzhou, Fujian, China; ^3^ The School of Public Health, Xiamen University, Xiamen, Fujian, China; ^4^ Department for Chronic and Noncommunicable Disease Control and Prevention, Fujian Provincial Center for Disease Control and Prevention, Fuzhou, Fujian, China; ^5^ Department of Breast Surgery, Shengli Clinical Medical College of Fujian Medical University, Fujian Provincial Hospital, Fuzhou, Fujian, China

**Keywords:** Europe burden of disease, breast cancer, chronic diseases, population attributable fraction, risk factors

## Abstract

**Background:**

Breast cancer (BC) represents a significant health challenge in Europe due to its elevated prevalence and heterogeneity. Despite notable progress in diagnostic and treatment methods, the region continues to grapple with rising BC burdens, with comprehensive investigations into this matter notably lacking. This study explores BC burden and potential contributing risk factors in 44 European countries from 1990 to 2019. The aim is to furnish evidence supporting the development of strategies for managing BC effectively.

**Methods:**

Disease burden estimates related to breast cancer from the Global Burden of Disease 2019(GBD2019) across Eastern, Central, and Western Europe were examined using Joinpoint regression for trends from 1990 to 2019. Linear regression models examined relationships between BC burden and Socio-demographic Index (SDI), healthcare access and quality (HAQ), and BC prevalence. We utilized disability-adjusted life year(DALY) proportions for each risk factor to depict BC risks.

**Results:**

In Europe, the BC burden was 463.2 cases per 100,000 people in 2019, 1.7 times the global burden. BC burden in women was significantly higher and increased with age. Age-standardized mortality and DALY rates of BC in Europe in 2019 decreased by 23.1%(average annual percent change: AAPC -0.92) and 25.9%(AAPC -1.02), respectively, compared to 1990, in line with global trends. From 1990 to 2019, age-standardized DALY declined faster in Western Europe (-34.8%, AAPC -1.49) than in Eastern Europe (-9.4%, AAPC -0.25) and Central Europe (-15.0%, AAPC -0.56). Monaco, Serbia, and Montenegro had the highest BC burden in Europe in 2019. BC burden was negatively correlated with HAQ. In addition, Alcohol use and Tobacco were significant risk factors for DALY. High fasting plasma glucose and obesity were also crucial risk factors that cannot be ignored in DALY.

**Conclusion:**

The burden of BC in Europe remains a significant health challenge, with regional variations despite an overall downward trend. Addressing the burden of BC in different regions of Europe and the increase of DALY caused by different risk factors, targeted prevention measures should be taken, especially the management of alcohol and tobacco should be strengthened, and screening services for BC should be popularized, and medical resources and technology allocation should be optimized.

## 1 Introduction

Cancer is one of the major chronic diseases that threaten the health of people all over the world (1, 2). Based on the Global Cancer Statistics of 2020, breast cancer has overtaken lung cancer as the primary global cancer incidence in 2020, registering an estimated 2.3 million new cases. It stands as the fifth most common cause of cancer deaths worldwide, with 685,000 fatalities. Among malignant tumors affecting females, breast cancer constitutes a quarter of all cancer cases and one-sixth of cancer-related deaths. In the majority of countries, breast cancer holds the top positions for both incidence and mortality rates (3). Faced with such a high incidence and mortality, it has brought great challenges for the prevention and control of breast cancer in numerous nations worldwide, especially for the more economically developed areas.

Europe accounts for 9.7% of the global population. It was reported that in 2020 there were nearly 580,000 new BC cases and 160,000 BC deaths in Europe (4). As one of the most economically developed regions, the medical level, social welfare, and per capita income of Europe are among the top in the world. In terms of BC prevention and control, Europe has explored and practiced different prevention and control models, which have played an effective role in reducing the overall disease burden of breast cancer. Up to now, no related studies have focused on the disease burden of breast cancer in Europe. So far, no research has been conducted in Europe regarding the burden of BC. Therefore, a comprehensive analysis of the overall disease burden of BC in Europe not only can provide a basis for prevention and control of BC burden but also holds significant reference value for regions with a higher global burden of BC.

Our study aimed to assess the burden of BC in Europe based on the latest estimates from the 2019 Global Burden of Disease (GBD) study. We estimated all measures related to disease burden, such as mortality rates, Years of Life Lost (YLLs), Years Lived with Disability (YLDs), and Disability-Adjusted Life Years (DALYs) for both genders at national and subnational levels. The evaluation covered age groups and the Socio-Demographic Index (SDI), highlighting the burden linked to known risk factors from 1990 to 2019 across a 30-year period.

## 2 Materials and methods

### 2.1 Data sources

We obtained data on the burden of breast cancer globally and for 44 European countries for 1990-2019 from the Global Health Data Exchange (GHDx) website (http://ghdx.healthdata.org/gbd-results-tool). The methodology for amalgamating GBD data has been extensively expounded in prior research (5, 6). In brief, GBD partners gathered data on cancer incidence cases from diverse cancer registries or compiled databases. The identification of cancer events was based on the International Classification of Diseases (ICD) version 10 codes about breast cancer (ICD-10: C50.0-C50.9). Mortality estimations were then derived from vital registration systems and cancer registry systems, computed using cancer-specific Cause of Death Ensemble models (CODEms), as detailed in other references (7). The estimation of DALYs involves two main components: YLLs and YLDs (8). YLLs are computed by multiplying the number of deaths by the remaining life expectancy, while YLDs are calculated by multiplying disease prevalence by corresponding disability weights and adjusting for comorbidities. All metrics were presented as counts, crude rates, and age-standardized rates.

The Socio-Demographic Index (SDI) reflects the developmental status, incorporating per capita income, total fertility rate (age <25 years), and average educational attainment (for those age ≥15 years), with values ranging from 0 to 1 (9). The Healthcare Access and Quality (HAQ) is the only indicator that uses risk-standardized mortality rates and mortality incidence rates to compare and estimate the accessibility and quality of healthcare across different countries or regions. The index spans from 0 (worst) to 100 (best) (10).

### 2.2 Countries in the European region

In the GBD 2019, Europe was divided into Eastern (Belarus, Estonia, Latvia, Lithuania, Moldova, Russia, and Ukraine), Central (Albania, Bosnia and Herzegovina, Bulgaria, Croatia, Czech Republic, Hungary, Montenegro, North Macedonia, Poland, Romania, Serbia, Slovakia, and Slovenia), and Western (Andorra, Austria, Belgium, Cyprus, Denmark, Finland, France, Germany, Greece, Iceland, Ireland, Israel, Italy, Luxembourg, Malta, Monaco, Netherlands, Norway, Portugal, San Marino, Spain, Sweden, Switzerland, and the UK), comprising a total of 44 countries (5).

### 2.3 Statistical analysis

We utilize the Average Annual Percent Change (AAPC) to assess the time trend of breast cancer burden from 1990 to 2019. The Joinpoint model was employed to calculate AAPC and its 95% confidence interval (95%CI). This model is based on segmental regressions established from the temporal characteristics of disease distribution, enabling trend fitting and optimization for each segment. Linear regression was used to calculate AAPCs and their 95%CI. The rates were transformed into a logarithmic scale and utilized as the dependent variable, while each year served as the independent variable in the analysis.

We used descriptive statistical analysis and data were expressed as frequency and proportions in tables. A linear regression model was utilized to explore the relationship between the age-standardized DALY rates for breast cancer in 44 European countries in 2019 and factors such as the SDI Index, HAQ Index, and breast cancer prevalence. Statistical significance was set at a two-sided P-value below 0.05. The study employed SPSS (version 24.0), Joinpoint (version 5.0.2), and R (version 4.2.1) for data analysis.

## 3 Results

### 3.1 Burden of breast cancer

**Figure 1:**
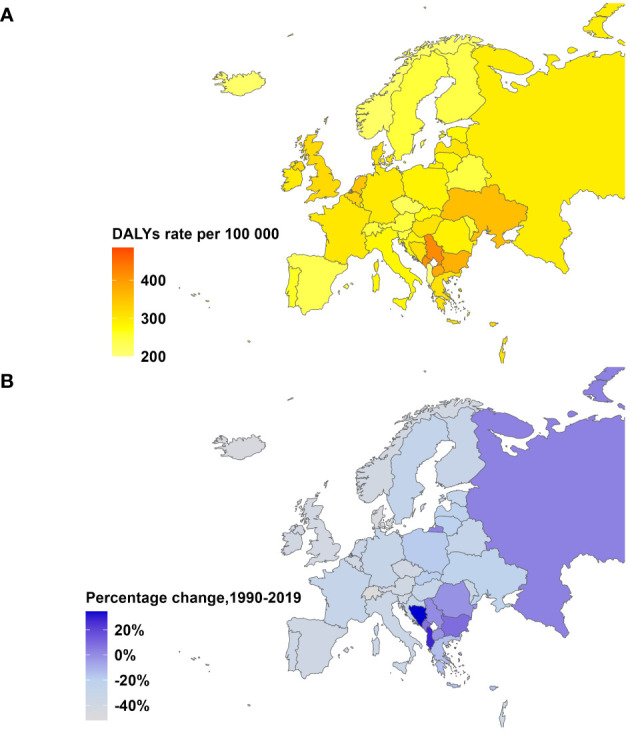
Age-Standardized DALYs Rate in 2019 and Percentage Change from 1990-2019 for breast cancer in Europe, both sexes. **(A)** Age-standardized DALYs rate per 100,000 people in 2019. **(B)** Percentage change in age-standardized DALYs rate, 1990-2019.

All-age DALYs for BC in Europe with percentage change from 1990 to 2019 were shown in **Table S2**. In 2019, the crude DALYs for breast cancer in Europe were 463.2 per 100,000 people (95% UI 430.8-498.0), which is 1.7 times higher than the global average. Age-standardized rate of death and DALYs for BC in Europe with Percentage Change and AAPC were demonstrated in **Tables 1** **,** **2** and **Figure 1**. In 2019, the age-standardized death rates and DALY rates for breast cancer in Europe were 10.8 cases and 291.4 cases per 100,000, respectively. These rates decreased by 23.1% (AAPC -0.92) and 25.9% (AAPC -1.02) compared to 1990, aligning with global trends. Compared to Eastern Europe (-9.4%, AAPC -0.25) and Central Europe (-15.0%, AAPC -0.56), Western Europe (-34.8%, AAPC -1.49) witnessed a faster decline in age-standardized DALYs for breast cancer from 1990 to 2019.

**Table 1:** Age-Standardized Rate of Death and DALYs for Breast Cancer in Europe, with Percentage Change: 1990 and 2019.

	Age-standardized death rate per 10,0000			Age-standardized DALYs rate per 10,0000		
	1990	2019	Percentage change 1990-2019	1990	2019	Percentage change 1990-2019
Global	9.8 (9.3, 10.2)	8.6 (7.9, 9.2)	-12.0% (-18.5, -6.1)	275.3 (263.2, 288.8)	247.6 (228.7, 266.1)	-10.1% (-17.3, -3.5)
Europe	14.1 (13.5, 14.4)	10.8 (9.9, 11.5)	-23.1% (-26.9, -18.8)	393.3 (381.7, 404)	291.4 (271.8, 313.2)	-25.9% (-29.8, -21.4)
Eastern Europe	11.0 (10.7, 11.4)	10.6 (9.2, 12.3)	-3.8% (-15.9, 11)	335.3 (325.4, 348)	303.9 (263.1, 354)	-9.4% (-20.8, 5.3)
Central Europe	12.4 (11.9, 12.7)	11.3 (9.9, 12.9)	-8.7% (-19.9, 3.4)	350.8 (340.8, 360.4)	298 (257.7, 344)	-15.0% (-26.1, -3.3)
Western Europe	16.2 (15.4, 16.6)	10.9 (10, 11.5)	-32.8% (-35.7, -30.2)	445.3 (430.6, 459.1)	290.5 (272.7, 311.1)	-34.8% (-37.6, -31.7)

**Table 2:** Average Annual Percent Change in the Age-Standardized Rates of Death and DALYs for Breast Cancer in Europe, 1990-2019.

	Age-standardized death rate			Age-standardized DALYs rate		
	AAPC (95%CI)	T value	P value	AAPC (95%CI)	T value	P value
Global	-0.43 (-0.51, -0.35)	-10.5432	<0.001	-0.35 (-0.42, -0.28)	-9.3145	<0.001
Europe	-0.92 (-1.02, -0.81)	-17.4041	<0.001	-1.02 (-1.2, -0.83)	-10.7889	<0.001
Eastern Europe	-0.04 (-0.61, 0.53)	-0.1505	0.88	-0.25 (-0.82, 0.33)	-0.8389	0.402
Central Europe	-0.29 (-0.47, -0.11)	-3.133	0.002	-0.56 (-0.74, -0.39)	-6.3505	<0.001
Western Europe	-1.38 (-1.5, -1.26)	-22.4589	<0.001	-1.49 (-1.55, -1.42)	-43.5883	<0.001

**Figure 2:**
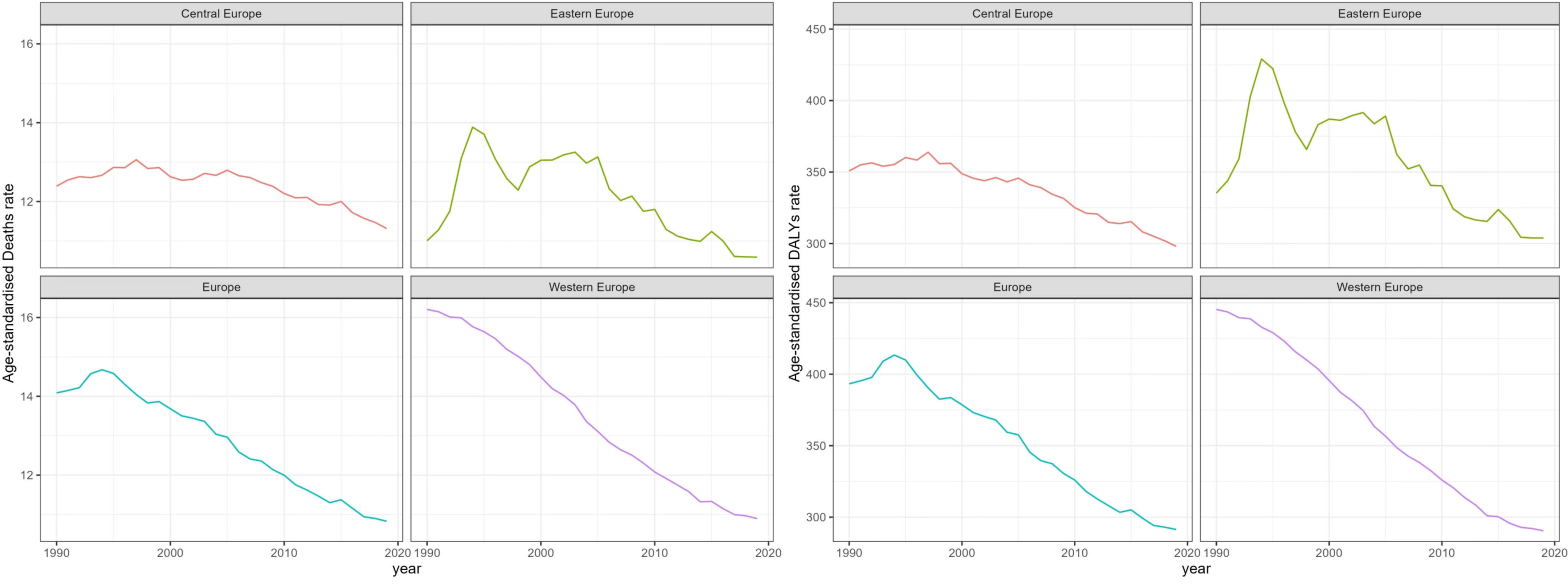
Trend of Age-Standardized death Rates and DALY Rates for Breast Cancer in Europe from 1990 to 2019.

Time trend of age-standardized death rates and DALY rates for BC in Europe from 1990 to 2019 were illustrated in **Figure 2**, **Supplementary Figures S1–S4.** During this period, Eastern Europe exhibited a fluctuating pattern in age-standardized death rates for breast cancer, with two distinct inflection points indicating increasing trends: 6.54 APC from 1990-1994 and 1.98 APC from 1998-2002, while other periods had decreasing trends. Meanwhile, Central Europe observed a gradual 8.7% decrease (AAPC -0.29) in age-standardized death rates for breast cancer from 1990 to 2019. In contrast, Western Europe experienced a significant annual decrease in age-standardized death rates, declining by 32.8% (AAPC -1.38). In 2019, Eastern Europe topped the rankings in Europe for age-standardized DALY rates for breast cancer at 303.9 individuals per 100,000 people (95% UI 263.1-354.0), with Ukraine and the Russian Federation bearing the highest burden. Across Europe, Monaco, Serbia and Montenegro in Central Europe bored the heaviest burden of breast cancer.

**Figure 3:**
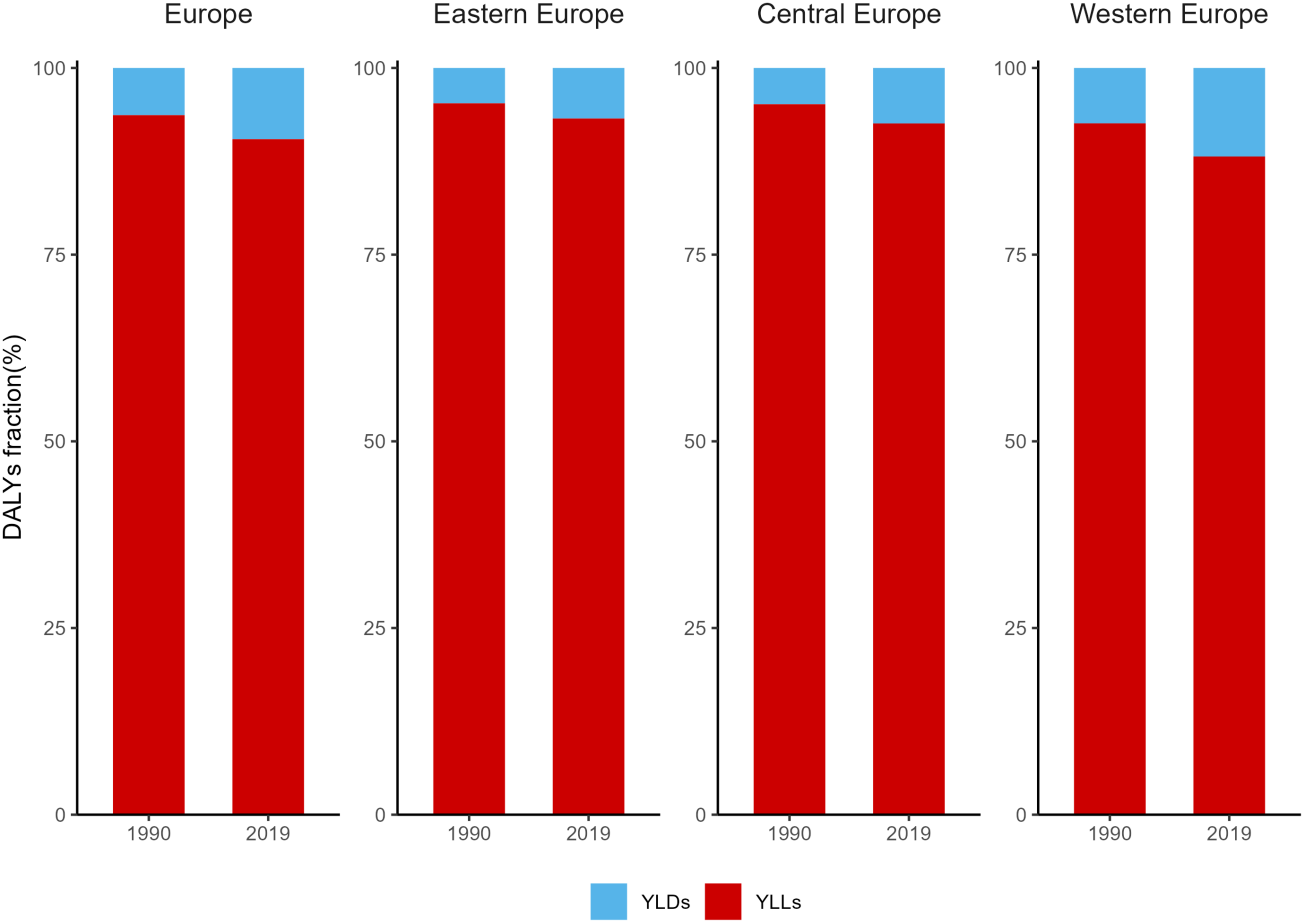
Fractions of YLDs and YLLs for Breast Cancer in Europe in 1990 and 2019. YLLs, Years of life lost; YLDs, years lived with disability.

The fractions of YLDs and YLLs for BC in Europe in 1990 and 2019 were demonstrated in **Figure 3**, **Supplementary Table S5**. In 1990, there was a significant disparity in the proportion of YLLs and YLDs in the burden of breast cancer in Europe, with YLLs accounting for 93.7% and YLDs for 6.3%. As of 2019, the YLLs showed a modest reduction due to the three regions reported an increase in the proportion of YLDs in DALYs during the period 1990-2019. Specifically, Western Europe had the highest YLD rates at 11.9%, with YLLs accounting for 88.1%. However, in Eastern Europe and Central Europe, YLLs still represented over 90%.

### 3.2 Age distribution of breast cancer burden

**Figure 4:**
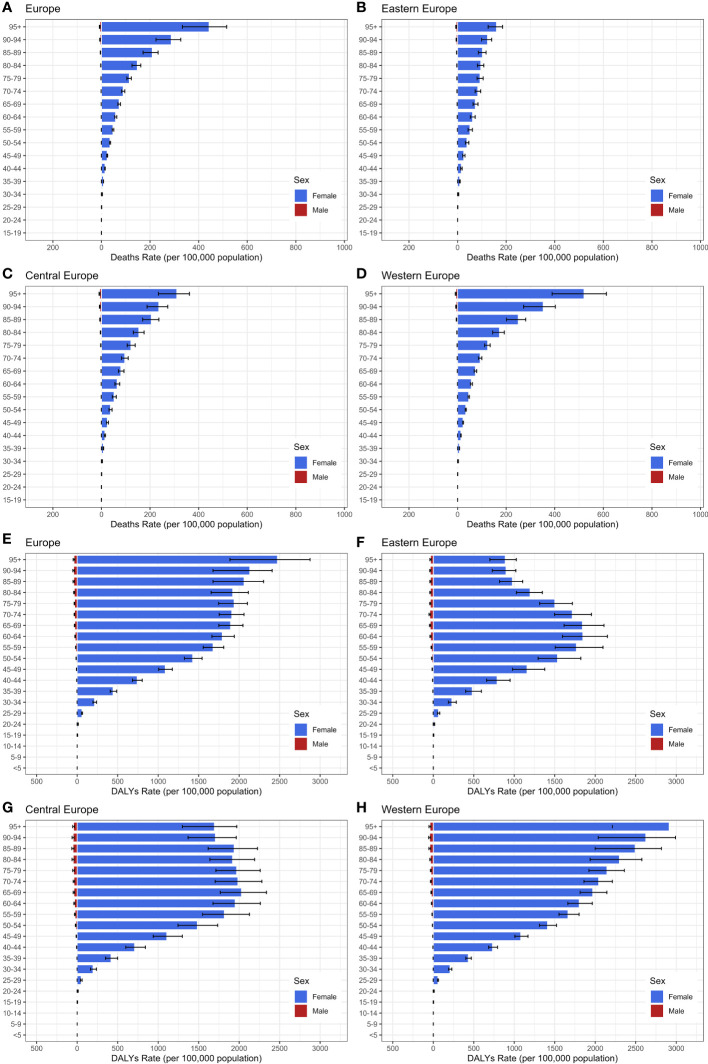
The age distribution of Age-Standardized Death Rate and DALYs Rate for Breast Cancer in 2019, categorized by gender. **(A–D)** age-standardized death Rate; **(E–H)**: age-standardized DALYs Rate.

**Figure 4** highlights significant gender disparities in the burden of breast cancer across Europe in 2019, with females exhibiting markedly higher rates compared to males. Moreover, both the age-standardized death rate and DALYs rate gradually increased with age. Concerning cancer mortality, Age-standardized death rates raised continuously with advancing age, with the 95+ age group being the highest for mortality in each European region. Regarding breast cancer DALYs among Eastern European females, absolute numbers increased with age up to 65 years, subsequently declining with further aging. Notably, the 50-79 age group carried the highest DALY rate, accounting for 60.3% of the total burden. In Central European women, a sharp escalation in DALYs rate was noted between the ages of 25 and 64, with the highest burden observed in the 60-89 age group, representing 47.0% of the overall DALYs burden. The DALYs rate for Western European women exhibited a consistent rise with advancing age, culminating in the most substantial burden observed within the 95+ age group, representing 12.2% of the total DALYs.

### 3.3 SDI, HAQ, and prevalence: correlation

**Figure 5:**
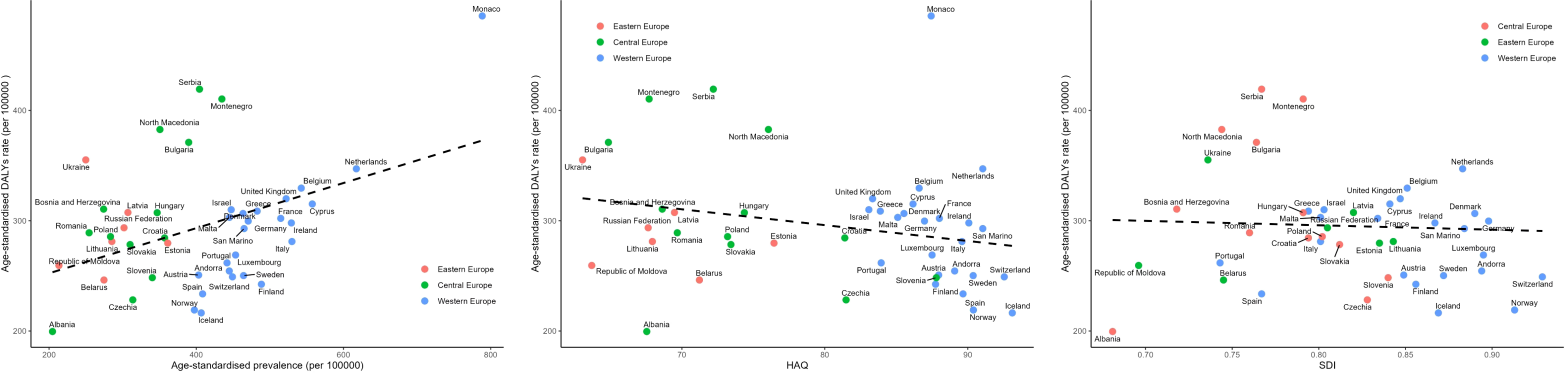
Correlation of SDI, HAQ, and Prevalence with Age-Standardized DALYs for Breast Cancer in Europe in 2019. SDI, the Socio-demographic Index; HAQ, the Healthcare Access and Quality Index.

The correlation of SDI, HAQ, and prevalence with age-standardized DALYs for BC in Europe in 2019 were presented in **Figure 5**, **Supplementary Table S6**. Linear regression analysis demonstrated a negative correlation (R^2 = ^0.127, P = 0.021) between age-standardized DALYs and HAQ. Higher HAQ values signified a lower burden of breast cancer, as demonstrated in Western Europe countries, which had high HAQ values and a relatively low burden of breast cancer. However, countries like Monaco, Netherlands, and Belgium exhibited significantly higher burdens than expected. There is no significant correlation between SDI, breast cancer prevalence and DALYs (R^2 = ^0.002, P = 0.782; R^2 = ^0.015, P = 0.499)

### 3.4 Risk factors

**Figure 6:**
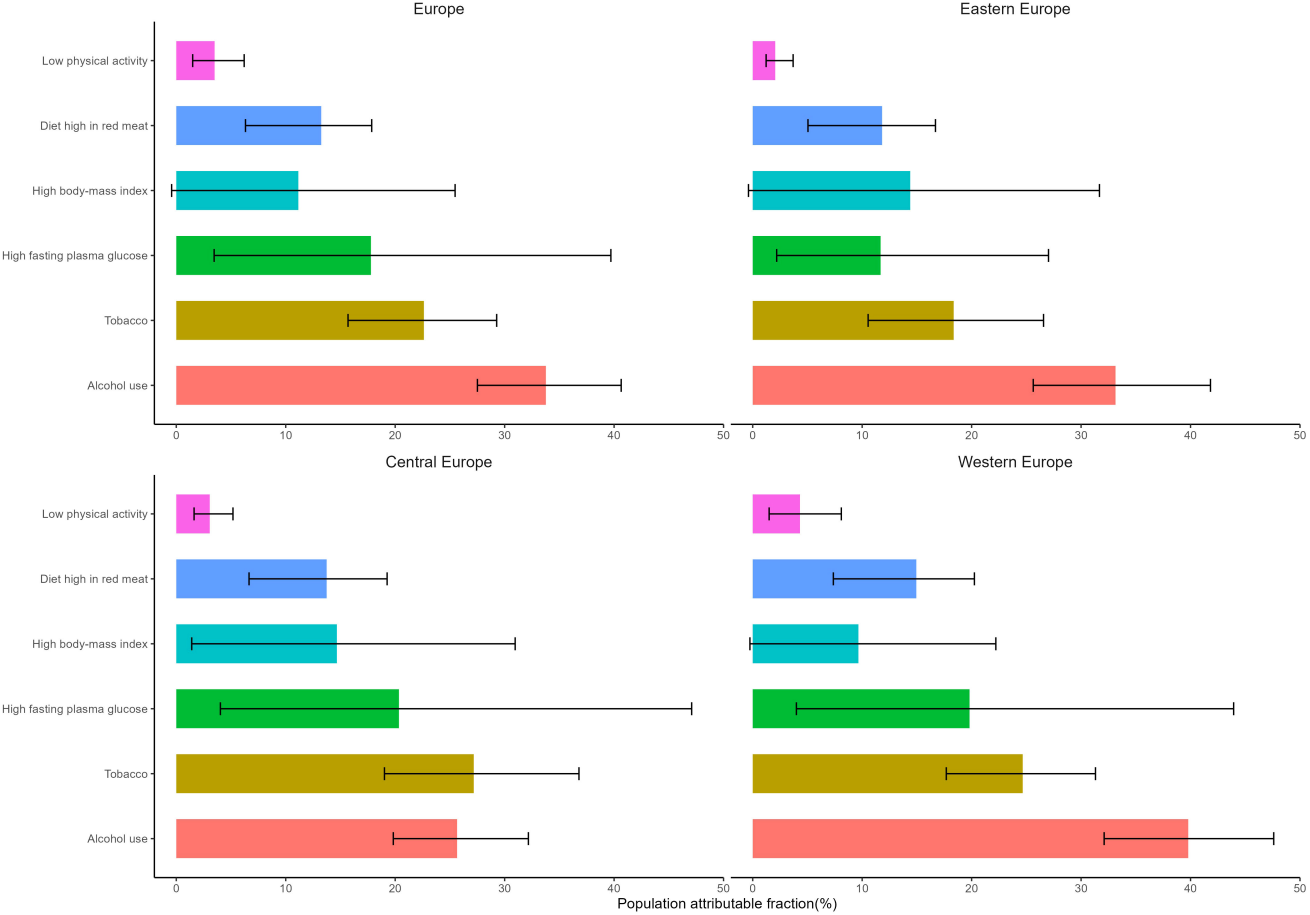
Proportion of Age-Standardized DALYs for Breast Cancer Attributable to Various Risk Factors in Europe in 2019.

The DALY proportions for each risk factor attributable to BC were illustrated in **Figure 6**, **Supplementary Tables S7–S8**. In 2019, results of risk factors attributable to breast cancer revealed that the proportion of DALY for Tobacco, Alcohol use, High fasting plasma glucose, High body-mass index, Diet high in red meat, and Low physical activity were 22.2%, 33.1%, 17.4%, 10.9%, 13.0%, and 3.4% respectively in Europe. Alcohol use emerged as a primary risk factor for the burden of breast cancer in Europe, with proportions exceeding 30% in both Eastern and Western Europe. Following closely was Tobacco, while Low physical activity had the smallest share. Particularly noteworthy is that in Central Europe, the proportion of Tobacco attributable to breast cancer was higher at 26.0%, surpassing Alcohol use at 24.5%.

## 4 Discussion

During the period from 1990 to 2019, both the age-standardized death rates and DALY rates of breast cancer in the European region were higher than the global average. In 2019, there was regional heterogeneity in the distribution of breast cancer burden among Eastern, Central, and Western Europe, with higher burdens observed in Eastern and Central Europe. Breast cancer primarily affects women in Europe, with variations in burden across different regions and age groups.

It is worth noting that in Europe, the age-standardized death rate for breast cancer is significantly lower than the age-standardized incidence rate. However, there is a significant difference in the distribution of age-standardized DALYs rates, with YLDs (9.5%) and YLLs (90.5%), which may be attributed to several factors: breast cancer tends to occur at younger ages compared to other chronic diseases, and the shorter survival time of breast cancer patients leading to premature deaths results in a loss of life expectancy. Additionally, this difference may also be related to the lower weighting assigned to breast cancer.

The research findings demonstrate a negative correlation between the HAQ and the burden of breast cancer in Europe. This indicates that in countries with higher healthcare quality and accessibility, the age-standardized DALY rates for breast cancer are lower. This evidence precisely explains the significant decrease in age-standardized breast cancer mortality rates and DALY rates in Western Europe. The high level of medical care in this region results in a concentration of breast cancer mortality among middle-aged and elderly populations, ultimately improving and extending the lifespan of breast cancer patients (11). In contrast, the burden of breast cancer in Eastern and Central Europe is concentrated among the middle-aged population, with a decrease in the burden among the elderly. This disparity may stem from insufficient awareness among the elderly regarding breast cancer risks and symptoms, leading to delays in diagnosis and treatment initiation. Diagnosis of advanced-stage breast cancer is more common among elderly women in these regions (12), potentially due to its more aggressive nature. Treatment goals for advanced-stage breast cancer shift towards prolonging life and enhancing the quality of life, rather than aiming for a cure. Additionally, older women in these regions may receive less intensive cancer treatments and exhibit poorer treatment compliance, resulting in higher mortality rates compared to younger female patients. Compared to Western Europe, healthcare service development levels in Eastern and Central European countries are relatively backward. This includes limited medical resources and unequal cancer care in Eastern and Central countries (13). For example, in Ukraine, where gaps exist in the breast cancer screening system and a lack of corresponding diagnostic and treatment follow-up (12), as well as in Serbia, where women under 50 undergo minimal breast cancer screening (14). To address the challenge of the burden of breast cancer in Europe, it is crucial to prioritize improving breast cancer treatment opportunities and standards in Central and Eastern Europe to reduce mortality rates in these regions (15). The advantages of the European cancer registry systems lie in monitoring breast cancer incidence and mortality rates to identify high-risk breast cancer populations for early diagnosis and treatment. Although the European cancer registry system covers 60% of the population (16), efforts should continue to expand screening programs and implement early interventions to reduce breast cancer treatment costs, further alleviating the burden of disease in Central and Eastern European countries.

In Europe, the main risk factors for breast cancer, ranked from high to low impact, include alcohol use, tobacco, high fasting plasma glucose(FPG), high red meat diet, high body mass index(BMI), and low physical exercise.

Alcohol use:

Alcohol use has emerged as a primary risk factor for breast cancer burden in Europe, with rates exceeding 30% in both Eastern and Western Europe. Public awareness of the link between alcohol consumption and breast cancer risk has been insufficient (17–19). Research indicates that female alcohol consumption, even at lower levels, is associated with an increased risk of breast cancer. Therefore, it is crucial to enhance awareness among women in Europe about the hazards of alcohol consumption in relation to breast cancer and to implement a series of policies and regulations, such as increasing alcohol taxes or restricting alcohol sales times or advertising, to prevent and reduce the incidence of breast cancer.

Tobacco:

Tobacco has been proven to be closely associated with an increased risk of developing breast cancer (20, 21). Analysis suggests that this association may be due to various reasons. Firstly, key components of tobacco promote cell proliferation, inhibit apoptosis, and create a favorable microenvironment for tumor growth, thereby promoting the development of breast cancer (22). Secondly, tobacco smoke can elevate the risk of breast cancer metastasis by inducing phenotypic transitions, acquiring self-renewing stem-like traits, chronic inflammation, and suppressing host immune defenses (23, 24). Several population-based studies have indicated that tobacco increases breast cancer-specific mortality rates and overall mortality rates (25, 26). Hence, there is a need to further raise awareness among healthcare professionals and the general public about the harms of tobacco, intensify smoking cessation campaigns, and expedite the enforcement of smoke-free legislation to protect women from the detrimental effects of tobacco.

High fasting plasma glucose, high red meat diet, high BMI, and low physical exercise

We have explored the primary risk factors contributing to the burden of breast cancer in Europe and found that high FPG ranks among the top three risk factors for BC DALYs, particularly in Western and Central Europe. Relevant research has shown that elevated plasma glucose levels can induce DNA repair enzyme damage through the production of free radicals, hindering deoxyribonucleic acid(DNA) repair and leading to cancer (27, 28). Additionally, high FPG increases the risk of postoperative BC recurrence. Furthermore, our study identified high red meat diet, high BMI, and lack of exercise as additional risk factors for the burden of breast cancer in Europe. Research indicates that the oxidative activity of heme iron and non-heme iron in a high-red meat diet, along with carcinogenic byproducts generated during high-temperature cooking processes, may be major factors contributing to breast cancer development. Patients with high BMI often exhibit enhanced aromatase enzyme activity, elevated estrogen levels, and suppression of breast cell apoptosis, consequently leading to the onset and progression of BC (29). Some unhealthy lifestyle factors, particularly obesity and high fasting plasma glucose, have been found to show an increasing trend in Europe over the past decade (30). Without proper control and management, obesity and high fasting plasma glucose may potentially become significant risk factors for breast cancer in the future. Based on these findings, conducting health education targeted at women is a crucial step in breast cancer treatment and prevention. Educating individuals about health can help shift their health perspectives. Referring to Mediterranean and Nordic dietary patterns (31), controlling energy intake, actively engaging in physical exercise, enhancing body function vitality and metabolic capacity, and maintaining a stable and healthy weight through a healthy diet and exercise can reduce the likelihood of developing breast cancer.

In summary, there are significant disparities in the burden of breast cancer in Europe: 1)Regional variations exist in the distribution of breast cancer disease burden. 2)Age-standardized DALYs for breast cancer differ among women of different age groups. 3)Variations exist in the composition of age-standardized DALY rates. Therefore, tailored prevention measures should be implemented to address the burden of breast cancer in different regions of Europe. There should be a particular focus on intensifying management efforts concerning risk factors such as alcohol and tobacco to reduce their impact on breast cancer incidence. Furthermore, early breast cancer screening services should be promoted, raising public awareness of breast cancer risks. It is essential to optimize healthcare resources and technological capabilities to ensure that patients receive timely and effective treatment.

## 5 Limitations

The study has limitations inherent in GBD research (5). In Europe, certain countries face significant shortages in obtaining accurate and high-quality data, forcing researchers to rely on data adjusted by covariates from other countries for analysis. This inevitably leads to bias in the data results. This phenomenon is particularly common when assessing the burden of breast cancer, both fatal and non-fatal. In the global burden of disease studies, we prioritize choosing data sources that are plentiful, accepting the impact of data adjustments, rather than relying solely on limited high-quality data sources. While using different definitions of breast cancer cases as adjustment strategies can increase available data resources, it also introduces measurement biases.

## Data availability statement

The original contributions presented in the study are included in the article/**Supplementary Material**. Further inquiries can be directed to the corresponding authors.

## Author contributions

SY: Writing – original draft, Writing – review & editing. XC: Writing – original draft, Writing – review & editing. XW: Writing – review & editing. XL: Writing – review & editing. SC: Writing – review & editing.
