# Supplementary material for: Disease burden of breast cancer and risk factors in Europe 44 countries, 1990-2019: findings of the global burden of disease study 2019
Source: Front Endocrinol (Lausanne). 2024 May 23;15:1405204. doi: 10.3389/fendo.2024.1405204 (PMC11153740; doi:10.3389/fendo.2024.1405204)
Supplement: Supplementary file 1 [file DataSheet_1.docx]

## **Supplementary Figures**

## **Figure S1 Joinpoint regression analysis of Age-standardized deaths rate of breast cancer at Global, Europe, Eastern Europe, Central Europe and Western Europe levels** **from 1990 to 2019.**


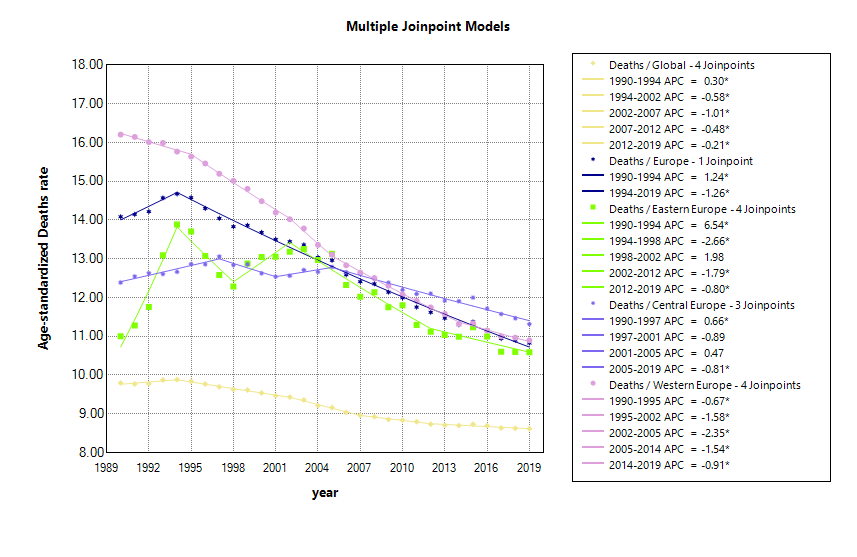


## **Figure S2 Joinpoint regression analysis of Age-standardized DALYs rate of breast cancer at Global, Europe, Eastern Europe, Central Europe and Western Europe levels from 1990 to 2019.**


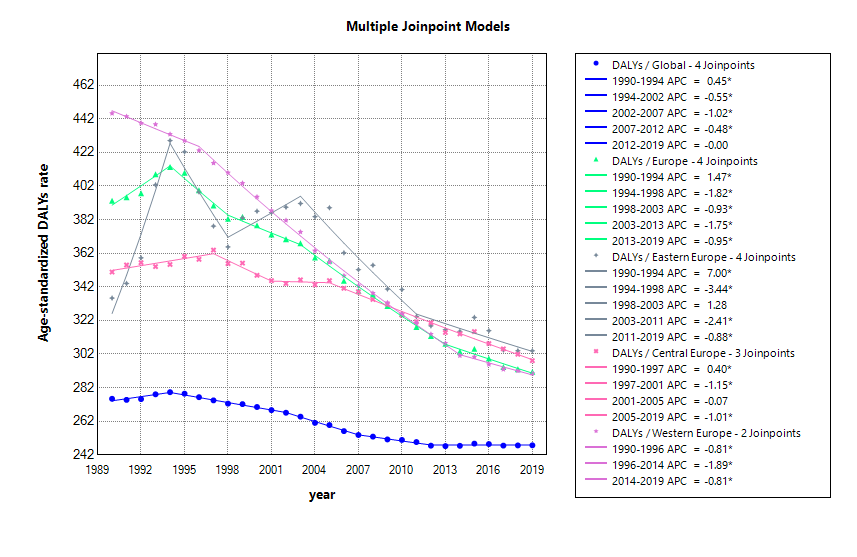


## **Figure S3 Joinpoint regression analysis of Age-standardized incidence rate of breast cancer at Global, Europe, Eastern Europe, Central Europe and Western Europe levels from 1990 to 2019.**


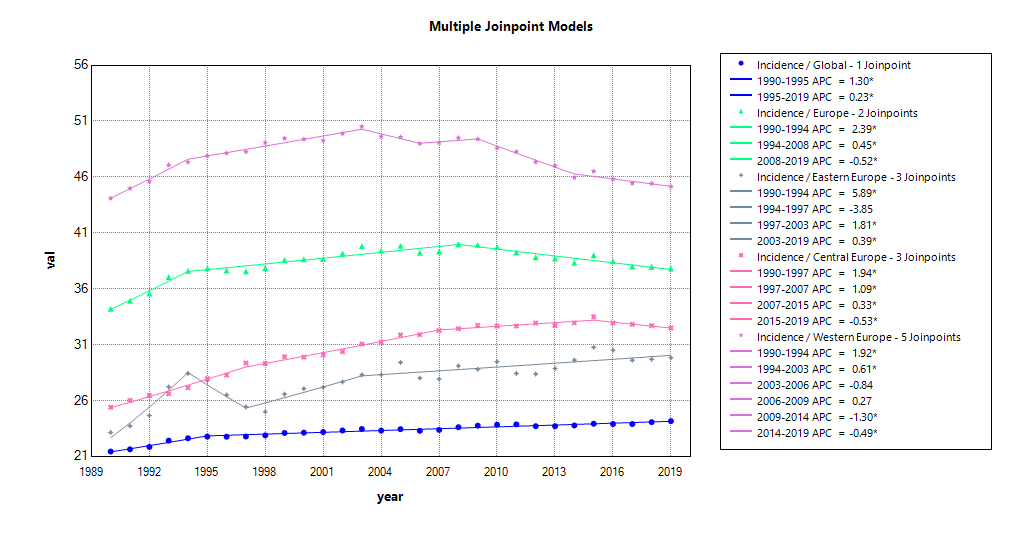


## **Figure S4 Joinpoint regression analysis of Age-standardized prevalence rate of breast cancer at Global, Europe, Eastern Europe, Central Europe and Western Europe levels from 1990 to 2019.**


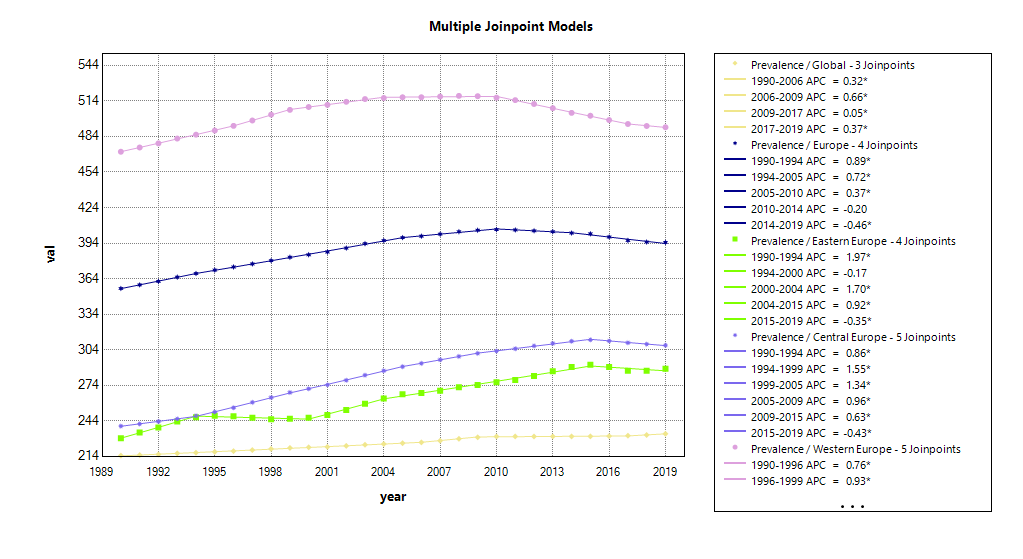


**1.2 Supplementary Tables**

## **Table S1 List of regions and countries of the Europe**

| Location | Countries of Europe |
| --- | --- |
| Eastern Europe | Republic of Moldova; Ukraine; Latvia; Russian Federation; Belarus; Lithuania; Estonia |
| Central Europe | Albania, Bosnia and Herzegovina, Bulgaria, Croatia, Czech Republic, Hungary, Montenegro, North Macedonia, Poland, Romania, Serbia, Slovakia, and Slovenia |
| Western Europe | Andorra, Austria, Belgium, Cyprus, Denmark, Finland, France, Germany, Greece, Iceland, Ireland, Israel, Italy, Luxembourg, Malta, Monaco, Netherlands, Norway, Portugal, San Marino, Spain, Sweden, Switzerland, and the UK |

## **Table S2 All-age DALYs for Breast Cancer in Europe, with Percentage Change: 1990 and 2019**

|  | All-age DALYs per 100000 | | |
| --- | --- | --- | --- |
|  | 1990 | 2019 | Percentage change  1990-2019 |
| Global | 218.3 (208.8, 229.2) | 266.6 (246.1, 286.6) | 22.1% (12.2, 30.9) |
| Europe | 476.4 (462.2, 489.6) | 463.2 (430.8, 498.0) | -2.8% (-7.9, 3.1) |
| Eastern Europe | 402.9 (391.1, 418) | 460.2 (400.1, 534.9) | 14.2% (0, 32.1) |
| Central Europe | 411.3 (399.7, 422.6) | 493 (429.2, 566.6) | 19.9% (4.6, 36.2) |
| Western Europe | 594.7 (573.5, 613.9) | 500.7 (465.9, 536.3) | -15.8% (-19.8, -11.8) |

|  | Prevalence(age-standardized rate) | | | Incidence(age-standardized rate) | | |
| --- | --- | --- | --- | --- | --- | --- |
|  | 1990 | 2019 | Percentage change 1990-2019 (%) | 1990 | 2019 | Percentage change 1990-2019 (%) |
| Global | 214.8 (198.7, 235.4) | 233.1 (214.4, 251.1) | 0.1 (0, 0.2) | 21.4 (20.6, 22.1) | 24.2 (22.1, 26.2) | 0.1 (0, 0.2) |
| Europe | 355.8 (328.7, 389.8) | 394.6 (360.5, 434.3) | 0.1 (0, 0.2) | 34.2 (33.3, 34.9) | 37.8 (33.8, 42.5) | 0.1 (0, 0.2) |
| Eastern Europe | 229.3 (212.2, 250.9) | 288.1 (252.2, 329.4) | 0.3 (0.1, 0.4) | 23.1 (22.5, 24) | 29.8 (25.5, 35.1) | 0.3 (0.1, 0.5) |
| Central Europe | 239.6 (222.8, 260.6) | 307.8 (273.7, 350) | 0.3 (0.1, 0.4) | 25.4 (24.6, 26.1) | 32.5 (28.1, 37.5) | 0.3 (0.1, 0.5) |
| Western Europe | 471.2 (433.8, 514.6) | 491.8 (438.9, 550.6) | 0 (-0.1, 0.2) | 44.1 (42.8, 45.1) | 45.1 (39, 51.8) | 0 (-0.1, 0.2) |

**Table S3 Age-Standardized Rate of Prevalence and Incidence for Breast Cancer in Europe, with Percentage Change: 1990 and 2019**

**Table S4 Age-Standardized Rate of Death and DALYs for Breast Cancer with Percentage Change: 1990 and 2019, in Europe, regions, and countries**

|  | Age-standardized death rate per 100000 | | | Age-standardized DALYs rate per 100000 | | |
| --- | --- | --- | --- | --- | --- | --- |
|  | 1990 | 2019 | Percentage change 1990-2019 (%) | 1990 | 2019 | Percentage change 1990-2019 (%) |
| Global | 9.8 (9.3, 10.2) | 8.6 (7.9, 9.2) | -12.0(-18.5, -6.1) | 275.3 (263.2, 288.8) | 247.6 (228.7, 266.1) | -10.1%(-17.3, -3.5) |
| Europe | 14.1 (13.5, 14.4) | 10.8 (9.9, 11.5) | -23.1(-26.9, -18.8) | 393.3 (381.7, 404) | 291.4 (271.8, 313.2) | -25.9(-29.8, -21.4) |
| Eastern Europe | 11.0 (10.7, 11.4) | 10.6 (9.2, 12.3) | -3.8(-15.9, 11) | 335.3 (325.4, 348) | 303.9 (263.1, 354) | -9.4(-20.8, 5.3) |
| Belarus | 10.9 (10.4, 11.4) | 8.4 (6.5, 10.8) | -22.9 (-40.6,-0.9) | 344.7 (328.5, 361.2) | 246.2 (187.5, 324.4) | -28.6 (-46.1,-5.5) |
| Estonia | 13.3 (12.5, 14.1) | 10.3 (8.1, 13) | -22.7 (-39.9,-1.8) | 388.6 (365.7, 413.3) | 279.7 (213.9, 359.7) | -28 (-45.2,-6.8) |
| Latvia | 12.9 (12.3, 13.6) | 11.2 (8.5, 14.4) | -13.6 (-34,11.9) | 392.5 (371.2, 415) | 307.5 (229.2, 406) | -21.7 (-41.8,3.1) |
| Lithuania | 11.4 (10.8, 12) | 10 (8, 12.2) | -12.5 (-29.6,7.2) | 353.6 (334.3, 373.1) | 281.2 (223.7, 351.1) | -20.5 (-36.8,-0.5) |
| Republic of Moldova | 11.8 (11.2, 12.4) | 9 (7.6, 10.5) | -24 (-35.2,-11.5) | 379.7 (360, 400.8) | 259.4 (220.8, 307.8) | -31.7 (-42,-19.6) |
| Russian Federation | 9.5 (9.2, 9.8) | 10.4 (8.6, 12.4) | 9.9 (-9.1,30.4) | 285.4 (276.8, 298.5) | 293.6 (243.3, 354.3) | 2.9 (-14.8,22.8) |
| Ukraine | 14.8 (14.1, 15.5) | 11.8 (9.4, 14.8) | -20 (-36.7,-0.7) | 458 (437.3, 479.6) | 355.2 (281.5, 447.1) | -22.5 (-38.9,-3) |
| Central Europe | 12.4 (11.9, 12.7) | 11.3 (9.9, 12.9) | -8.7(-19.9, 3.4) | 350.8 (340.8, 360.4) | 298 (257.7, 344) | -15.0(-26.1, -3.3) |
| Albania | 5.1 (4.7, 5.6) | 6.5 (4.8, 8.7) | 26.6 (-6.7,70.8) | 155.5 (143, 169) | 199.6 (145.6, 271.4) | 28.3 (-7.1,77.6) |
| Bosnia and Herzegovina | 7.7 (7.1, 8.4) | 11.7 (9.2, 14.7) | 52.1 (18.2,91.7) | 230.8 (213, 251.2) | 310.5 (240.6, 401) | 34.5 (1.7,72.5) |
| Bulgaria | 10.8 (10, 11.5) | 12.9 (10.2, 16) | 19.5 (-5.8,50.8) | 341.1 (317.1, 366.1) | 371.1 (287.5, 473.3) | 8.8 (-16.3,40.1) |
| Croatia | 14.7 (13.6, 15.9) | 11.6 (9.1, 14.3) | -21.2 (-38.3,-1.4) | 391.8 (362.3, 424.7) | 284.4 (219.3, 361.5) | -27.4 (-44.3,-6.4) |
| Czechia | 14.3 (13.7, 14.9) | 9.2 (7.6, 11.1) | -35.6 (-47,-21.9) | 379.3 (363.4, 395.4) | 228.2 (184.7, 279.5) | -39.8 (-51.1,-26.3) |
| Hungary | 15.7 (15.1, 16.3) | 11.8 (9.6, 14.2) | -25.2 (-38.4,-10.2) | 433 (414.8, 450.1) | 307.3 (248.8, 376.4) | -29 (-42.9,-13) |
| Montenegro | 13.3 (11.1, 16.3) | 14.6 (11.9, 17.6) | 9.1 (-17.5,38.6) | 382 (314.5, 472.1) | 410.4 (327.9, 505.2) | 7.4 (-18.9,38) |
| North Macedonia | 12.4 (11.1, 14) | 14.1 (11, 17.6) | 13.1 (-14.7,45.6) | 390.6 (350.5, 435.6) | 382.7 (291.1, 488) | -2 (-27.3,28.5) |
| Poland | 12.6 (12, 12.9) | 11.1 (8.9, 13.8) | -11.8 (-28.8,8.9) | 349.4 (339.7, 359.5) | 285.6 (229.8, 356.7) | -18.3 (-34.4,1.6) |
| Romania | 9.6 (9.2, 10) | 10.4 (8.5, 12.6) | 8.6 (-10.9,31.5) | 294.7 (282.6, 309.1) | 289.1 (233.2, 355.4) | -1.9 (-20.2,20.7) |
| Serbia | 14.7 (12.9, 16.7) | 16 (12.8, 19.9) | 9.3 (-15.1,40.6) | 418.7 (372.8, 472) | 419.3 (330.5, 531.4) | 0.1 (-23.9,29.7) |
| Slovakia | 11.8 (11.1, 12.5) | 10.9 (8.2, 14) | -7.7 (-29.9,20.4) | 344.3 (321.6, 367.3) | 278.4 (207.6, 368.5) | -19.1 (-39.8,8.4) |
| Slovenia | 15 (11.6, 19.3) | 10 (7.8, 13.1) | -33.4 (-53.3,-5.4) | 408.7 (307.7, 537.4) | 248.4 (191.7, 328.1) | -39.2 (-58.4,-10.8) |
| Western Europe | 16.2 (15.4, 16.6) | 10.9 (10, 11.5) | -32.8(-35.7, -30.2) | 445.3 (430.6, 459.1) | 290.5 (272.7, 311.1) | -34.8(-37.6, -31.7) |
| Andorra | 10.4 (7.8, 14.2) | 9 (6.5, 12) | -13.1 (-44.5,26.1) | 282.1 (209.1, 386.8) | 254.5 (183.1, 345.1) | -9.8 (-42.3,32.1) |
| Austria | 15.2 (14.3, 15.7) | 9.8 (9, 10.6) | -35.2 (-39.6,-30.4) | 414.6 (395.2, 433.5) | 250.8 (230.5, 271.3) | -39.5 (-43.9,-34.7) |
| Belgium | 20.3 (19.2, 21.2) | 12.7 (11.5, 13.7) | -37.6 (-41.8,-32.9) | 548.7 (524.4, 572) | 329.6 (302.9, 362.1) | -39.9 (-44.7,-34.9) |
| Cyprus | 13.8 (12, 16) | 12.2 (10.6, 13.9) | -11.8 (-27.9,6.4) | 368.8 (316.5, 429.6) | 315.2 (268.2, 368.2) | -14.5 (-30.9,5.2) |
| Denmark | 21.9 (20.9, 22.7) | 12.3 (11, 13.5) | -43.6 (-48.5,-38.4) | 601.5 (578.1, 627.6) | 306.6 (275.7, 339.8) | -49 (-53.8,-43.9) |
| Finland | 12.4 (11.8, 12.9) | 8.9 (8, 9.7) | -28.5 (-34.3,-22.1) | 348.4 (331.8, 366.3) | 242.4 (218.3, 269.8) | -30.4 (-37.2,-23.1) |
| France | 15.8 (14.9, 16.5) | 11.2 (10, 12.1) | -29.6 (-35.1,-24.2) | 425.6 (407.1, 442.6) | 302.1 (274.5, 332.2) | -29 (-34.9,-22.5) |
| Germany | 15.8 (15, 16.4) | 11.5 (10.5, 12.4) | -26.9 (-32,-21.7) | 428.6 (410.7, 445.7) | 299.7 (275, 326) | -30.1 (-35.4,-24.2) |
| Greece | 12.6 (11.9, 13.1) | 11.7 (10.7, 12.7) | -6.6 (-13.9,1) | 360.4 (343.9, 379.1) | 308.7 (283.5, 337.6) | -14.3 (-21.3,-6.6) |
| Iceland | 14.1 (13, 15.3) | 7.7 (6.7, 8.7) | -45.2 (-52.2,-37.8) | 401.2 (369, 435) | 216.4 (189.1, 242.7) | -46.1 (-53.1,-38.4) |
| Ireland | 17.9 (16.9, 18.7) | 11 (9.9, 12) | -38.5 (-43.6,-32.2) | 506.2 (482, 531.3) | 298 (269.1, 332.5) | -41.1 (-47,-34.3) |
| Israel | 17.4 (16.4, 18.2) | 11.8 (10.7, 12.7) | -32 (-37.4,-26.7) | 490 (467.9, 513.7) | 310 (284.5, 337) | -36.7 (-42.2,-31.6) |
| Italy | 14.8 (14.1, 15.1) | 10.2 (9.2, 10.9) | -30.6 (-34.6,-27) | 423.1 (407.5, 439.6) | 281.1 (260.6, 302.8) | -33.5 (-37,-29.9) |
| Luxembourg | 19 (17.6, 20.3) | 10.4 (8.9, 12.2) | -45.4 (-52.7,-36.6) | 505 (468.5, 539.5) | 268.9 (230.9, 312.4) | -46.7 (-54.3,-38.1) |
| Malta | 19.1 (17.7, 20.5) | 11.1 (9.6, 12.8) | -42.1 (-50.4,-33) | 518.1 (481.1, 555.3) | 303 (263.6, 350.4) | -41.5 (-49.5,-32.2) |
| Monaco | 18.7 (17.7, 19.5) | 17.4 (12.9, 21.7) | 2.5 (-25.7,41.9) | 468.3 (356.2, 605.3) | 485.7 (357.5, 631.2) | 3.7 (-26.4,46.6) |
| Netherlands | 13.7 (13, 14.2) | 13.2 (11.9, 14.3) | -29.8 (-35.1,-24.3) | 514.2 (492.3, 536.2) | 347.1 (316.8, 380.8) | -32.5 (-38,-26.4) |
| Norway | 14.3 (13.7, 15) | 8.3 (7.5, 9) | -39.3 (-43.2,-34.7) | 375.3 (359.5, 389.8) | 219.1 (201.5, 240.6) | -41.6 (-45.5,-36.8) |
| Portugal | 12.9 (12.1, 13.4) | 9.3 (8.5, 10.2) | -34.9 (-40.4,-28.9) | 409.6 (392.6, 426.6) | 261.8 (238.9, 290) | -36.1 (-41.9,-29.2) |
| San Marino | 12 (11.3, 12.5) | 10.5 (6.8, 15.8) | -5.4 (-42.5,49.2) | 283 (232, 345) | 292.9 (189.4, 452.9) | 3.5 (-38.2,66.4) |
| Spain | 19.4 (18.2, 20.2) | 8.4 (7.6, 9.1) | -34.8 (-39.5,-30.2) | 378.4 (362.4, 394.3) | 233.7 (213.4, 255.8) | -38.2 (-43,-33) |
| Sweden | 20.6 (19.6, 21.1) | 9.4 (8.6, 10.2) | -38.2 (-43,-33) | 342.5 (326.7, 358.2) | 250.3 (229.8, 270.6) | -26.9 (-32.2,-21.4) |
| Switzerland | 16.9 (12.9, 21.4) | 9.6 (8.5, 10.5) | -50.2 (-54.3,-45.9) | 517.3 (492.9, 541.1) | 249.1 (224.8, 275) | -51.8 (-56.1,-47) |
| United Kingdom | 11.1 (9.2, 13.4) | 12.3 (11.4, 12.9) | -40.2 (-42.5,-37.8) | 548.8 (531.6, 566.1) | 319.9 (302.3, 340.9) | -41.7 (-44.1,-38.9) |

**Table S5 Fractions of YLDs and YLLs for Breast Cancer in Europe in 1990 and 2019.**

|  | YLLs(%) | | YLDs(%) | |
| --- | --- | --- | --- | --- |
|  | 1990 | 2019 | 1990 | 2019 |
| Global | 94.5% | 93.2% | 5.5% | 6.8% |
| Europe | 93.7% | 90.5% | 5.5% | 9.5% |
| Eastern Europe | 95.3% | 93.2% | 4.7% | 6.8% |
| Central Europe | 95.1% | 92.6% | 4.9% | 7.4% |
| Western Europe | 92.6% | 88.1% | 7.4% | 11.9% |

**Table S6 Correlation of SDI, HAQ, and Prevalence with Age-Standardized DALYs for Breast Cancer in Europe in 2019. SDI, the Socio-demographic Index; HAQ, the Healthcare Access and Quality Index.**

|  | R^2^ | F | P |
| --- | --- | --- | --- |
| SDI | 0.002 | 0.077 | 0.782 |
| HAQ | 0.127 | 5.817 | 0.021 |
| Prevalence | 0.015 | 0.467 | 0.499 |

**Table S7 Proportion of Age-Standardized DALYs for Breast Cancer Attributable to Various Risk Factors in Europe in 2019**

|  | Tobacco | Alcohol use | High fasting plasma glucose | High body-mass index | Diet high in red meat | Low physical activity |
| --- | --- | --- | --- | --- | --- | --- |
| Europe | 22.2% | 33.1% | 17.4% | 10.9% | 13.0% | 3.4% |
| Eastern Europe | 20.1% | 36.2% | 12.8% | 15.7% | 12.9% | 2.2% |
| Central Europe | 26.0% | 24.5% | 19.4% | 14.0% | 13.1% | 2.9% |
| Western Europe | 21.8% | 35.2% | 17.5% | 8.5% | 13.2% | 3.8% |

**Table S8 Proportion of Age-Standardized DALYs for Breast Cancer Attributable to Various Risk Factors in Europe in 2019**

|  |  | 1990 | | 2019 | | % change(1990-2019) | |
| --- | --- | --- | --- | --- | --- | --- | --- |
| Location | Risk factors | both | female | both | female | both | female |
| Global | Tobacco | 19.1 (12.5,25.7) | 36.9 (23.9,49.8) | 12.7  (7.5,17.5) | 24.6  (14.4,34) | -33.4 (-42.7,-26.2) | -33.4 (-42.6,-26.2) |
|  | Alcohol use | 20.1 (16.6,23.9) | 38.1 (31.4,45.2) | 13.1 (10.6,15.6) | 24.4 (19.7,29.2) | -35.1 (-38.4,-31.4) | -36.1 (-39.3,-32.2) |
|  | High fasting plasma glucose | 12.2 (2.3,27.2) | 22.7 (4.2,51.1) | 14.9 (2.9,33.5) | 28.5 (5.5,64) | 22.9 (13.3,34.1) | 25.4 (15.6,36.9) |
|  | High body-mass index | 7.9 (1.6,17) | 14.2 (2.5,31.1) | 11.2 (3.5,21.4) | 21 (6.3,40.6) | 42.8 (5.9,169.2) | 48.2 (7.3,194.3) |
|  | Diet high in red meat | 9.7 (4.8, 12.9) | 18.6 (9.2,24.7) | 7.8 (3.7,10.4) | 14.9 (7.1,19.9) | -19.8 (-25.9,-13.7) | -19.8 (-26.2,-13.5) |
|  | Low physical activity | 2.8 (1.3,5.0) | 5.1 (2.4,9.4) | 2.4 (1.2,4.2) | 4.6 (2.3,8) | -12.7 (-20.4,-2.3) | -11.2 (-19.3,-0.6) |
| Europe | Tobacco | 33.9 (23.9,44.2) | 63.8 (45.2,82.9) | 22.6 (15.7,29.3) | 42.7 (29.7,55.4) | -33.4 (-37.4,-29.1) | -31.9 (-36,-27.6) |
|  | Alcohol use | 49.6 (40.8,58.7) | 59.2 (47.7,70.9) | 33.8 (27.5,40.6) | 62.7 (51.1,75.5) | -31.9 (-36.1,-27.4) | -30.8 (-35,-26.2) |
|  | High fasting plasma glucose | 16.3 (3.1,36.7) | 28.5 (5.3,63.9) | 17.8 (3.5,39.7) | 32.1 (6.3,72) | 25.5 (10.5,45.1) | 14.4 (7.7,23.8) |
|  | High body-mass index | 10.2 (-2.2,24.8) | 16.9 (-5.4,40.4) | 11.2 (-0.4,25.5) | 18.9 (-2.7,44.9) | 76.1 (-626.6,554.7) | 21.7 (-220.9,232.3) |
|  | Diet high in red meat | 19.4 (9.7,25.8) | 28 (13.1,36.8) | 13.2 (6.3,17.9) | 24.6 (11.7,33.2) | -26.1 (-42.9,-10.4) | -30.5 (-36.4,-25.1) |
|  | Low physical activity | 4.3 (1.9,7.8) | 5.5 (3.1,9.6) | 3.5 (1.5,6.2) | 6.3 (2.7,11.4) | 3.5 (-12.7,20.4) | -15.2 (-21.9,-7.4) |
| Eastern Europe | Tobacco | 19.1 (9.9,27.4) | 34 (17.7,48.9) | 18.4 (10.5,26.6) | 32.9 (18.9,47.7) | -3.7 (-18.7,18.9) | -3.2 (-18.3,19.9) |
|  | Alcohol use | 33.8 (27.3,40) | 60.2 (48.6,71.4) | 33.1 (25.6,41.8) | 58.9 (45.6,74.6) | -1.8 (-16.9,17.4) | -2.2 (-17.6,17.5) |
|  | High fasting plasma glucose | 9.3 (1.7,21.5) | 15.4 (2.8,35.8) | 11.7 (2.2,27) | 19.7 (3.7,45.6) | 25.5 (10.5,45.1) | 28 (12.7,48.2) |
|  | High body-mass index | 8.2 (-4.9,21.8) | 9.7 (-12.3,31.7) | 14.4 (-0.4,31.7) | 21.6 (-4.3,50.1) | 76.1 (-626.6,554.7) | 123.1 (-875.6,1048.5) |
|  | Diet high in red meat | 16 (7.7,21.1) | 27.9 (13.6,36.9) | 11.8 (5,16.7) | 20.7 (9,29.3) | -26.1 (-42.9,-10.4) | -25.7 (-42.5,-9.6) |
|  | Low physical activity | 2 (1.3,3.6) | 3.3 (2.1,6.1) | 2.1 (1.2,3.7) | 3.4 (2.1,6.2) | 3.5 (-12.7,20.4) | 4.4 (-12,22) |
| Central Europe | Tobacco | 34 (24.1,44.1) | 83.1 (60.6,105) | 27.2 (19,36.8) | 51.5 (36,69.7) | -20.1 (-31.6,-7.8) | -19.2 (-30.8,-6.7) |
|  | Alcohol use | 32 (25.8,38.3) | 125.6 (103.4,149.2) | 25.7 (19.8,32.2) | 47.7 (36.8,59.8) | -19.7 (-31.2,-7) | -19.4 (-31.1,-6.4) |
|  | High fasting plasma glucose | 16 (3,35.8) | 35.5 (6.7,79.8) | 20.3 (4,47.1) | 36.8 (7.3,85.3) | 27.2 (10.7,47.2) | 29.3 (12.5,49.8) |
|  | High body-mass index | 10.5 (-1.8,24.2) | 18.8 (-3.8,46.2) | 14.7 (1.4,31) | 25.2 (0.8,54.7) | 39.8 (-125.2,242.7) | 49.7 (-215.3,433.1) |
|  | Diet high in red meat | 15.1 (7.1,19.8) | 44.5 (22.5,59.3) | 13.7 (6.6,19.3) | 25.7 (12.4,36.1) | -9 (-21,7) | -8.4 (-20.7,7.9) |
|  | Low physical activity | 3.1 (1.7,5.3) | 10.7 (3.9,20.6) | 3.1 (1.6,5.2) | 5.5 (2.9,9.4) | -1 (-18.4,13.5) | -0.4 (-17.8,14.7) |
| Western Europe | Tobacco | 43.8 (31.9,55.4) | 62.7 (44,81.8) | 24.7 (17.7,31.3) | 47.8 (34.4,60.8) | -43.7 (-46.7,-40.2) | -42.4 (-45.6,-38.9) |
|  | Alcohol use | 67 (55.1,79.6) | 90.5 (74.6,107.2) | 39.8 (32.1,47.6) | 75.9 (61.4,90.8) | -40.6 (-44.1,-37.1) | -39.5 (-43.1,-35.9) |
|  | High fasting plasma glucose | 20 (3.8,44.9) | 28.1 (5.2,63.5) | 19.8 (4,43.9) | 37.1 (7.5,82.5) | -1 (-6.7,7.6) | 4.5 (-1.6,13.8) |
|  | High body-mass index | 11.4 (-1.1,27) | 15.6 (-6.6,40) | 9.7 (-0.3,22.2) | 17.4 (-1.5,40.9) | -15.5 (-34.4,13.7) | -7.3 (-77.9,47.3) |
|  | Diet high in red meat | 23.8 (12.1,31.7) | 35.4 (17.6,47) | 14.9 (7.4,20.3) | 28.6 (14.2,38.9) | -37.1 (-40.8,-33.7) | -35.7 (-39.7,-32.2) |
|  | Low physical activity | 5.9 (2.2,11.3) | 7.5 (3.4,13.8) | 4.3 (1.5,8.1) | 8.1 (2.8,15.4) | -27 (-33.1,-20.4) | -24.1 (-30.9,-16.4) |
